# Proteins and antibodies in serum, plasma, and whole blood—size characterization using asymmetrical flow field-flow fractionation (AF4)

**DOI:** 10.1007/s00216-018-1127-2

**Published:** 2018-05-29

**Authors:** Mats Leeman, Jaeyeong Choi, Sebastian Hansson, Matilda Ulmius Storm, Lars Nilsson

**Affiliations:** 1 SOLVE Research & Consultancy AB, Medicon Village, 22381 Lund, Sweden; 2 Department of Food Technology, Engineering and Nutrition, Faculty of Engineering LTH, Lund University, 22100 Lund, Sweden

**Keywords:** Whole blood, Antibodies, Plasma, Serum, Asymmetric flow field-flow fractionation (AF4), Fluorescence labelling

## Abstract

The analysis of aggregates of therapeutic proteins is crucial in order to ensure efficacy and patient safety. Typically, the analysis is performed in the finished formulation to ensure that aggregates are not present. An important question is, however, what happens to therapeutic proteins, with regard to oligomerization and aggregation, after they have been administrated (i.e., in the blood). In this paper, the separation of whole blood, plasma, and serum is shown using asymmetric flow field-flow fractionation (AF4) with a minimum of sample pre-treatment. Furthermore, the analysis and size characterization of a fluorescent antibody in blood plasma using AF4 are demonstrated. The results show the suitability and strength of AF4 for blood analysis and open new important routes for the analysis and characterization of therapeutic proteins in the blood.

**Electronic supplementary material:**

The online version of this article (10.1007/s00216-018-1127-2) contains supplementary material, which is available to authorized users.

## Introduction

Protein-based drugs are a fast-growing sector within the pharmaceutical industry and are nowadays used in the treatment of numerous diseases. Therapeutic proteins are complex with respect to their large molecular size (cf. small-molecule drugs) and their secondary and tertiary structures that must be maintained to function effectively as drug molecules. These intrinsic properties of proteins are, together with environmental stress, part of the reasons that proteins are prone to aggregate in pharmaceutical processing, formulation, and during storage. The formation of protein aggregates can result in reduced drug efficacy and/or immunogenicity, which compromises patient safety [1]. Hence, control and elimination of protein aggregates are crucial part of the formulation of protein products.

Analyzing protein aggregates can be challenging due to the wide size range—from small oligomers to large sub-visible particles or even visible precipitation [2]. The primary method for determining size and amount of sub-visible aggregates is size-exclusion chromatography (SEC) in combination with suitable detectors. However, in recent years, SEC, although being well established, has been questioned as it can give erroneous estimates of the aggregate levels [3–5]. Regulatory authorities, such as the US Food and Drug Administration (FDA), now recommend conducting several complementary, orthogonal methods to verify the measurements. Analyses of protein aggregates in the finished formulation are important but even more central would be to understand the aggregation potential after administration, including interactions with blood components [6]. Analyzing the aggregate formation of protein therapeutics after drug administration to the patient without tampering with the extracted sample has long been sought for and can only be achieved by analyzing blood either in vivo or ex vivo.

Blood plasma is a protein-rich solution in which white and red blood cells, as well as platelets, are suspended, and serum is the remaining fluid after removal of the clot from whole blood with principally the same composition as plasma with the exception that the fibrinogens and clotting factors are absent. The protein concentration in plasma/serum is approximately 60–80 mg/mL of which about 50–60% are albumins and 40% globulins (10–20% immunoglobulin G, IgG) [7, 8]. The size distribution of blood components ranges from small molecules and ions (< 1 nm) to about 15 μm for white blood cells. Due to the complex nature and the large size range of components in blood, such samples are difficult to analyze and extensive sample pre-treatment is generally included. Typical sample preparation steps involve centrifugation, extraction, and filtration [9, 10]. This pre-treatment can, however, cause unintended artifacts such as unwanted loss of components, contamination, and protein aggregation. Therefore, it is highly desirable to be able to analyze and characterize proteins in blood with a minimization of change in conditions, i.e., maintaining physiological pH and salinity and avoiding surfactants and organic solvents.

In the FDA industry guidance on aggregate analysis, no specific analytical method is recommended [5]. However, thorough assessment using qualified methods is requested to eliminate the presence of aggregates and one referred to the method is asymmetrical flow field-flow fractionation (AF4). AF4 is a method in which separation is achieved by applying an external field (cross flow) in a ribbon-like open channel without a stationary phase [11–13]. Due to the absence of a stationary phase, several problems related to SEC are alleviated including minimization of non-specific protein adsorption, structural deformation at the surface and high shear forces which may result in degradation of analytes. Therefore, AF4 is a highly powerful technique that is increasingly being used for the separation and characterization of biomacromolecules and pharmaceutical molecules [14–16]. It has been proven to be a potential tool for studying biological structures such as proteins, antigens, and antibodies [17–21]. In previous studies, field-flow fractionation (FFF) has been utilized for the separation and characterization of blood plasma and lipoproteins [22–24]. Li et al. studied the possibility of separating lipoproteins in blood plasma using symmetrical flow FFF, Mädorin et al. focused on the interaction between a low molecular weight drug and plasma, and quantitative analysis by the recovery of the drug after fractionation by AF4, and the study by Park et al. study focused on comparing the plasma proteins (lipoproteins and albumin) between patients and healthy persons using frit-inlet AF4. However, in all cases, the plasma samples were prepared by some preparation methods (centrifugation and additives were added).

In this study, the purposes are to investigate the possibility to utilize AF4 for high-resolution separation of proteins and other components in serum and plasma and furthermore to separate whole blood without sample pre-treatment such as centrifugation and filtration. Furthermore, we investigate the feasibility of selectively separating and detecting a fluorescent antibody in the matrix.

## Materials and methods

### Materials

The salts used for carrier preparation (sodium chloride, di-sodium phosphate, potassium phosphate, potassium phosphate, potassium chloride, and sodium azide) were all analytical grade (Sigma-Aldrich, St Louis, MS, USA). The water was purified on a Millipore Plus unit (Merck Millipore, Darmstadt, Germany). The myoglobin, bovine serum albumin, and immunoglobulin G reference samples were obtained from Sigma-Aldrich. The human serum (order number H4522) was obtained from Sigma-Aldrich. The plasma was from rat and whole blood from mouse (kindly donated by Redoxis AB, Lund, Sweden). The FITC (fluorescein isothiocyanate)-labeled goat anti-human IgG antibody was obtained from Capra Science Antibodies, Ängelholm, Sweden. The FITC loading was estimated to 6.2/antibody.

### Method

The asymmetrical flow field-flow fractionation (AF4) analysis was performed on an Eclipse II (Wyatt technology, Dernbach, Germany) in connection with a 1100-series LC-system consisting of an ERC-3415 vacuum degasser (ERC), a G1311A pump, a G1329A auto sampler, a G1315A diode array U*V*/VIS detector, and a G1321C fluorescence detector (Agilent Technologies, Santa Clara, CA, USA). A Dawn Heleos II multi-angle light scattering (MALS) and Optilab t-Rex differential refractive index (dRI) detector were connected on-line (Wyatt technology) after the channel. The UV detector was monitored at 250 and 280 nm, the fluorescence detector was set to an excitation wavelength of 495 nm and monitoring the emission at 525 nm, and the MALS utilized a laser with 658 nm wavelength and measured scattered light with 17 detectors in the aqueous carrier liquid. The dRI detector operated at a wavelength of 658 nm.

Data collection was performed by Astra 6.2 (Wyatt technology). The asymmetrical flow field-flow fractionation channel was a Wyatt SC channel fitted with a 350-μm wide spacer. For the analyses, a 10- or 100-kDa regenerated cellulose (RC) membrane (Merck Millipore) was used. The carrier consisted of phosphate-buffered saline (PBS), pH 7.4 with 3 mM sodium azide (added to prevent microbial activity). Fractionation was run at ambient temperature (approximately 22 °C) and all experiments were repeated at least two times for reproducibility.

Performance testing of the AF4 separation as well as checking the MALS-RI detection and molar mass determination was done by analyzing solutions of myoglobin, bovine serum albumin, and immunoglobulin G. For the MALS data evaluation, the Zimm method was used and the dRI with a refractive index increment, dn/dc, of 0.185 mL/g for concentration determination to obtain molecular weight (MW). The AF4 separation method used a detector flow rate of 0.50 mL/min, giving a system pressure of approximately 3–4 bar depending on detector configuration. Before injection was started, the system was allowed to stabilize crossflows and pressures for 2 min. Injection flow rate was 0.2 mL/min, injection time 1 min, and the focusing time 2 min; crossflow during injection and focusing was the same as used during elution (2.0 mL/min). During elution, the crossflow rate, *Q*_*c*_, was 2.0 mL/min, which was kept constant for 4 min after the onset of elution, thereafter decaying according to Eq. 1

1 $$ {Q}_c={Q}_{c,0}\bullet {2}^{-\left(\raisebox{1ex}{$t$}\!\left/ \!\raisebox{-1ex}{${t}_{\frac{1}{2}}$}\right.\right)} $$

where *Q*_*c*,*0*_ is the volumetric crossflow rate at the onset of the decay, *t* is the time, and *t*_*½*_ is the decay rate (4 min in the present study). When the crossflow rate reached 0.15 mL/min, it was kept constant for the remainder of the separation.

Injection volume was 10 μL for all tests. Both blood serum, blood plasma, and whole blood were diluted 100-fold with the carrier (PBS) prior to injection onto the AF4-channel and the size separation. Assuming that the protein content of the serum or plasma is approximately 70 mg/mL, this gives that the protein concentration of the sample going onto the channel is approximately 700 μg/mL and the sample load on the AF4 channel is approximately 7 μg. For the tests with the whole blood, a 100-kDa molecular weight cutoff (MWCO) membrane was used to reduce the protein load on the channel by removing proteins with lower MW (such as serum albumin), which can exit the size separation channel through the membrane.

## Results and discussion

### Analysis of blood serum

**Fig. 1:**
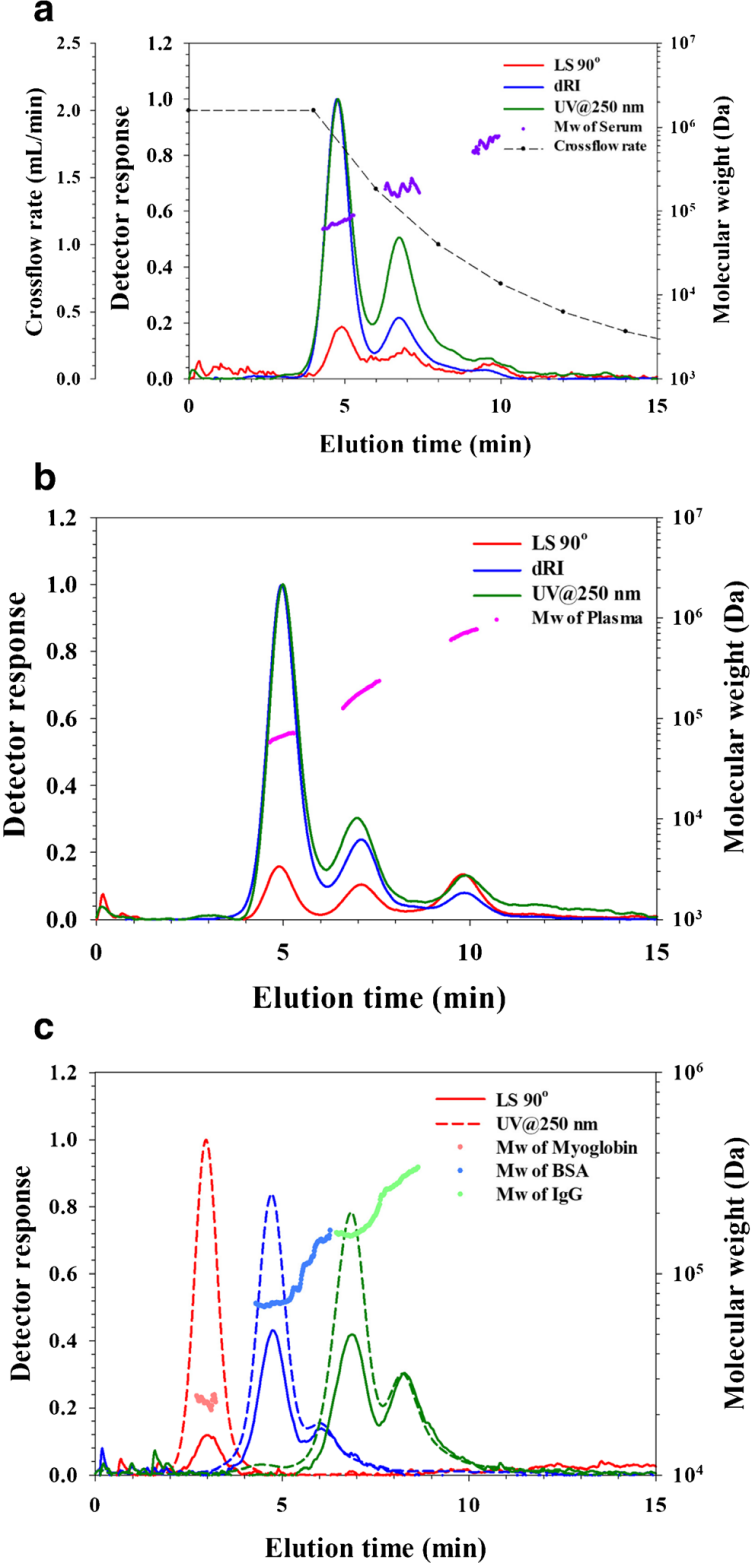
AF4-UV-MALS-dRI fractograms and molecular weight. UV trace at 250 nm (green), dRI trace (blue), and LS at 90° trace (red). **a** Analysis of blood serum. Injection volume was 10 μL of a 100× diluted serum sample, i.e., corresponding to 0.1 μL serum. **b** Analysis of plasma. Injection volume was 10 μL of a 100× diluted plasma sample, i.e., corresponding to 0.1 μL plasma. **c** Analysis of 17 kDa myoglobin (red trace), 67 kDa bovine serum albumin (blue trace), and ~ 150 kDa immunoglobulin G (green trace). The BSA and IgG samples contain dimers

Blood serum was diluted 100-fold with phosphate-buffered saline (PBS) (10 μL serum to which was added 990 μL PBS) prior to AF4 separation. The dilution was in order to reduce the viscosity of the solution. The elution time of the serum component was compared to the elution time obtained when analyzing the proteins myoglobin, bovine serum albumin, and immunoglobulin G using identical AF4 conditions (Fig. 1).

The serum sample shows a broad and multi-modal size distribution as detected by the UV, MALS, and dRI detectors. The peak with the maximum at 4.8 min in the serum sample in Fig. 1a corresponds well with the elution time of bovine serum albumin monomer peak in Fig. 1c (similar in size to human serum albumin), and the peak at 6.8 min corresponds well with the elution time obtained when analyzing immunoglobulin G (monomer peak in Fig. 1c). Further, the molar mass data from the MALS give the molar mass at peak apexes as 70 kDa (at 4.8 min) and 158 kDa (at 6.8 min). Two of the most abundant proteins in blood serum are expected to be serum albumin and IgG (based on literature values [7], often given as approximately 40 and 10 mg/mL, respectively). From these comparisons and based on the molecular weight data, we conclude that with the very high likelihood, the component eluting at 4.8 min is mainly serum albumin and the components eluting around 6.8 min are mainly IgG.

Obviously, given the huge number of different proteins that are to be expected to be present in blood serum, it can be expected that a large number of (similarly sized) proteins and other serum components are co-eluting with serum albumin and IgG. However, serum albumin and IgG are the most abundant protein and protein classes to be expected in blood and is likely the most significant contributors to the detected peaks.

The identity of the serum components eluted after IgG (8–11 min in Fig. 1a) is unknown but serum is known to contain proteins larger than IgG such as alpha-2-macroglobulin (720 kDa, ~ 3 mg/mL in serum) and IgM (950 kDa, ~ 1 mg/mL in serum) [25]. Furthermore, there is the possibility that some of the detected components are smaller proteins that are aggregated or associated with other proteins, making their size larger (thereby eluting later) than the individual monomer protein would.

### Analysis of blood plasma

Blood plasma was analyzed using the same settings as for the blood serum. The elution profile (Fig. 1b) is similar to that obtained for blood serum with components detected at similar elution time as serum albumin and immunoglobulin G. The most noticeable difference between the serum and plasma elution profile is that there is a larger amount of components eluted in the elution time range from 3 to 6 min (higher intensity of the peak at 4–6 min in the plasma sample) in Fig. 1b. It may be speculated that this may be due to fibrinogen (340 kDa protein) which is expected to be present in plasma but should not be present in serum (removed by centrifugation when the blood has been clotted). This results show a higher resolution for separation of blood plasma with FFF techniques, and more sensitive detection than the results from previous studies [22–24].

### Fluorescently labeled antibody in PBS and plasma

**Fig. 2:**
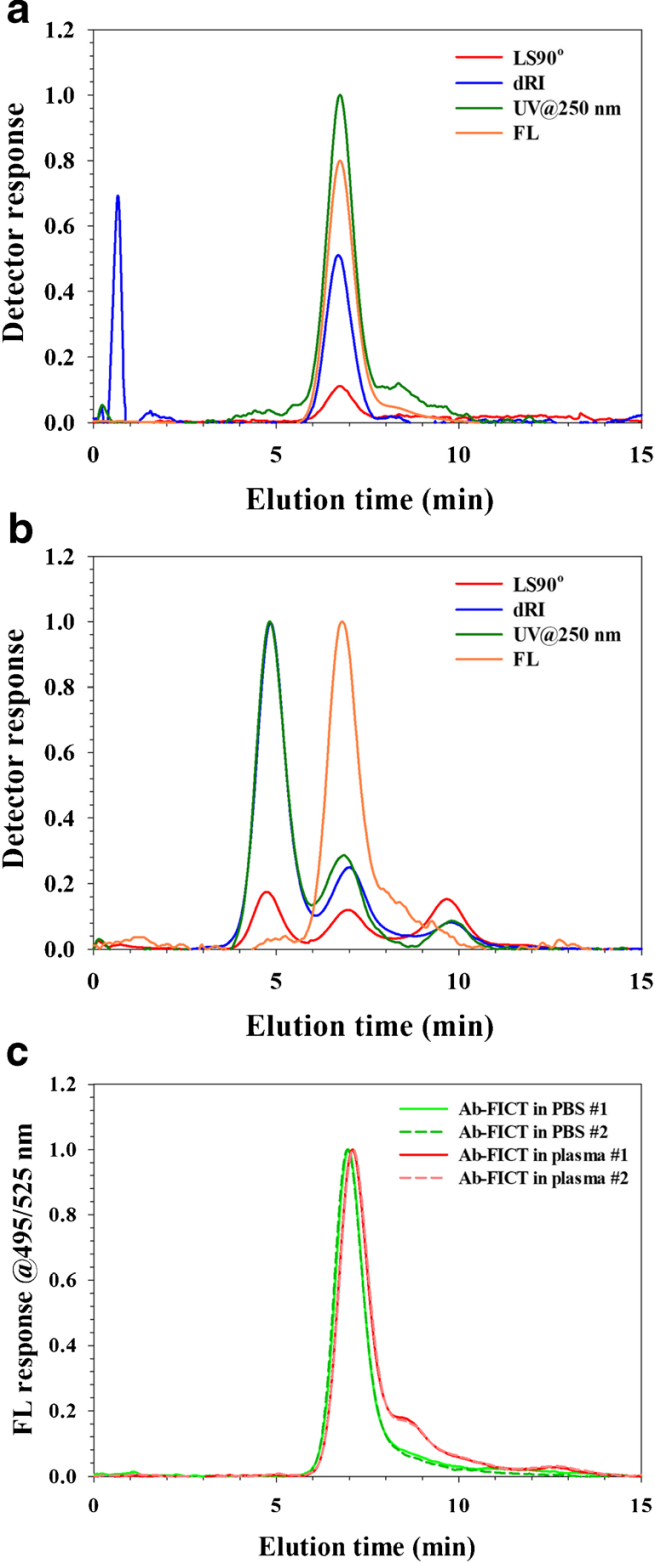
AF4-UV-MALS-dRI-FL fractograms. Detected by UV at 250 nm (green trace), MALS (red trace), FL at 495/525 nm (orange trace), and dRI (blue trace). **a** Analysis of fluorescently labeled antibody in PBS. The injection volume was 10 μL of a 100 μg/mL solution (mass load = 1 μg). **b** Analysis of fluorescently labeled antibody in blood plasma. The plasma was spiked for a concentration of 10 μg of fluorescently labeled antibody/mL plasma. The spiked plasma was then diluted 100× with PBS carrier before analysis (sample volume was 10 μL, corresponding to a mass load of 1 ng of fluorescently labeled antibody on channel). **c** Analysis of fluorescently labeled antibody in PBS (green trace) and in blood plasma (red trace). The plasma was spiked for a concentration of 100 μg of fluorescently labeled antibody/mL plasma. The spiked plasma was then diluted 100× with PBS carrier before analysis (sample volume was 10 μL, corresponding to a mass load of 10 ng of fluorescently labeled antibody on channel)

Both serum and plasma contain a wide variety of antibodies (estimated as > 10^7^ different antibodies [26]. Of the immunoglobulin G, there are four classes (IgG1–IgG4), each class in turn consisting of a huge range of antibodies often differing only very slightly in size and molar mass. To physically size separate those is not feasible with AF4 due to insufficient resolution. Thus, the antibodies eluting from the AF4 will elute as a mixture of many antibodies. Therefore, to be able to detect and monitor one specific type of antibody, a selective detection is needed. Fluorescence detection can offer such a selective detection if the antibody of interest is fluorescently labeled. To investigate if a fluorescently labeled antibody could be monitored in blood plasma, a goat antibody of the IgG type was utilized, which was labeled with the fluorescent marker FITC. For reference, the fluorescently labeled antibody was analyzed in PBS at a concentration of 100 μg/mL, sample volume 10 μL, to allow detection by UV, MALS, and dRI (Fig. 2a). The labeled antibody elutes at an elution time of 6.75 min similar to that of IgG in serum and plasma (6.8 min). It is noted that there is a shoulder on the peak (8–9 min) which is interpreted as the detection of (incompletely resolved) dimers.

The fluorescently labeled antibody was spiked into the plasma for a concentration of 10 μg/mL plasma and analyzed. The fluorescence detector detects the labeled IgG (Fig. 2b) while the UV, MALS, and dRI detectors detect all the other components of the plasma. At the spiked concentration (10 μg/mL plasma), the amount of labeled IgG on the channel is 1 ng, which is much too low mass for the UV, MALS, or dRI detectors to detect.

Comparing in more detail the elution profile of FICT-labeled IgG in PBS and in plasma reveals that there are differences as monitored by the fluorescence detector (Fig. 2c). The FICT-labeled IgG in PBS (green traces in Fig. 2c) has its apex at 6.75 min and shows a tail on the main peak (at 8–9 min) interpreted as a dimer (as noted above). In comparison, when the FICT-IgG is spiked into plasma (red trace in Fig. 2c), the peak shifts its apex to 6.9 min. Furthermore, the shoulder (dimer) is much more pronounced (higher intensity) when the sample is in plasma. The data is obtained on the same equipment, analyzed next to each other, in duplicate, at two different occasions, using the same conditions for both plasma and PBS. When the Ab-FICT is analyzed together with the plasma components, it is evident that both the main peak shift, indicating that it is acting as it was slightly larger during separation, and there is more increase in the antibody having a size similar to that of an antibody dimer. The conclusion is that interaction occurs between plasma components and the FICT-labeled IgG. Further investigations are required to elucidate the nature of the interaction.

### Analysis of whole blood

Fresh blood from mouse was obtained in order to investigate the capability of AF4 to separate an even more challenging matrix than shown above (i.e., including blood cells). The time between sampling from mouse and analysis was kept short (approximately 30 min) to minimize hemolysis. EDTA was added to prevent clotting. The blood was diluted 100-fold with the carrier (PBS) immediately before the analysis and the blood was then directly injected onto the AF4-channel. For these analyses, a 100-kDa membrane was utilized to remove lower MW proteins from the channel resulting in a lowering of the protein load on the channel. Note that these separations were performed on a different membrane, giving different channel thicknesses and thereby different elution times compared to the above reported results.

**Fig. 3:**
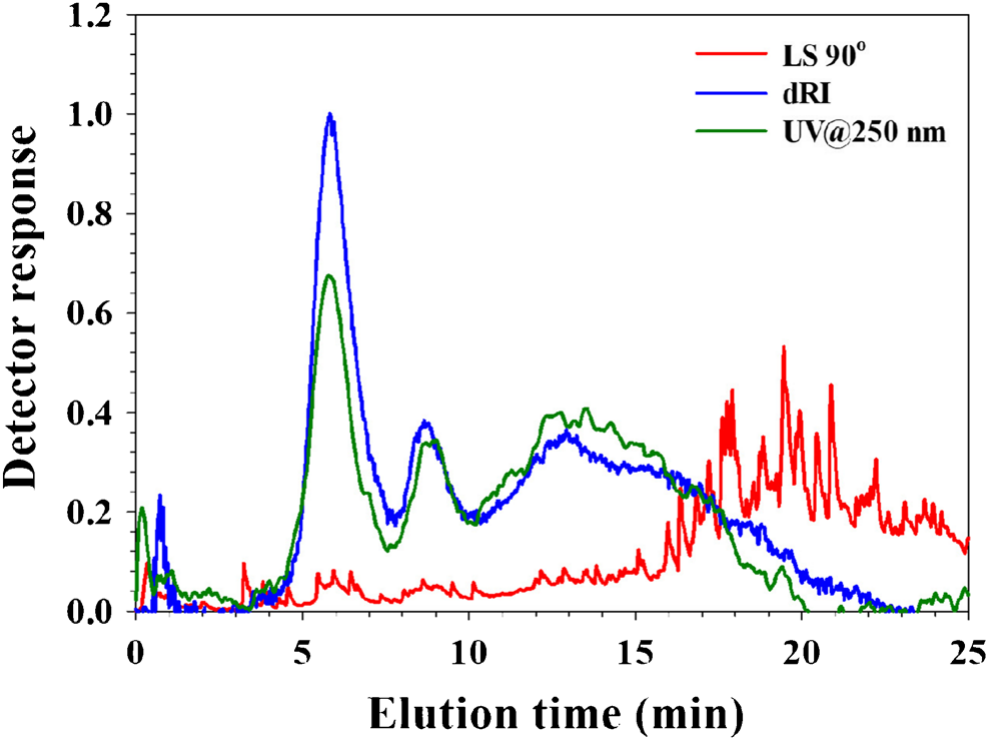
Analysis of whole blood by AF4-UV-MALS-dRI. UV trace at 250 nm (green), dRI trace (blue), and LS at 90° trace (red). Injection volume was 10 μL of a 100× diluted blood sample, i.e., corresponding to 0.1 μL whole blood

The fractogram (Fig. 3) shows a peak (at 6.0 min) that coincides with that of IgG and a peak at 8–10 min. In contrast to the data from serum and plasma, there is also a high abundance of larger sized components eluting in the time range from 10 to 20 min of which the identity is unknown. The light scattering signal shows multiple narrow peaks with high intensity (noise). Presumably, this is due to the elution of large, very high MW components or particulates of whole blood which is relatively few in number (such as entire blood cells or fragments of them).

Red blood cells have a size of approximately 0.5 × 8 μm and are outside the range of Brownian mode AF4 [27, 28]. As the intention of the analyses was not to separate these large components, it is not expected to have them properly separated. Rather, the objective was to test if it would be possible to inject a blood sample with a minimum of sample pre-treatment and without removing any of the components. The only perturbations to the blood are that EDTA has been added (to prevent clotting) and that the sample was diluted with the carrier (PBS) prior to injection (to reduce viscosity). No filtering, centrifugation, or addition of solvent modifiers has been done and the blood is analyzed in a PBS carrier which has an ionic strength and pH very similar to blood. To the extent, if whole blood can be analyzed by AF4, this test shows that it can be done; IgG and other proteins were eluted and the whole blood matrix did not cause the size separation to fail.

However, the red blood cells due to their color could be followed visually as they were injected onto the channel and it was noted that they stick to the membrane (see Electronic Supplementary Material (ESM) Fig. S1). After the analysis, the channel was rinsed (pumping the PBS carrier through the channel at 0.5 mL/min, no crossflow) and after approximately 1 h, no red traces could be visually observed in the channel. This is interpreted as that either (a) the cells has been flushed out or (b) the cells has been hemolysed and thereby lost their color (fragments perhaps being flushed out). Obviously, if whole blood would be analyzed routinely, it would require membrane type and chemistry, carrier composition, and rinsing protocols to be investigated and optimized. Furthermore, the potential of clogging the flow lines, both in the autosampler and the detectors, is a concern, although no clogging was observed during these duplicate analyses.

## Conclusions

The three most common types of blood samples (serum, plasma, and whole blood) were successfully size separated by AF4. The ability to analyze the blood samples under close to native conditions (i.e., ionic strength, pH, no filtering, centrifugation, the addition of organic modifiers/solvents, or detergents) opens the possibility for:

- Blood protein size profiling—investigating differences in the size distribution of blood between samples and changes over time
- Investigations of therapeutic proteins (fluorescently labeled) when in blood.

It is common to study therapeutic proteins in the formulation, for example, to investigate if aggregation or degradation occurs, usually as part of quality control or during formulation development. However, the possibility to use AF4 for size separation in blood makes aggregation or degradation behavior possible to be studied in the medium which the therapeutic protein should be present (i.e., blood). In this study, it is shown that it is possible to analyze an antibody in blood plasma. The results will aid in addressing the question on what happens with therapeutic proteins after they have been administrated [29]. In the light of this, it is possible that AF4 is the only presently available method for ex vivo analysis of protein aggregates (< 1 μm in size), which may cause immunogenicity, requiring a minimum of sample pre-treatment. The requirements for such studies are that a selective detection technique can be employed, such as fluorescence (by introducing a fluorescent label on the compound of interest). Mass spectrometry is another interesting technique to be used for the selective detection of therapeutic proteins separated as described in this paper which would not make fluorescent labelling necessary. The fluorescence labelling would be attractive to avoid as it introduces change in chemical properties of the protein which, in turn, could influence the propensity to form aggregates.

## Electronic supplementary material

ESM 1 (PDF 127 kb)

## References

[1] Frokjaer S, Otzen DE (2005). Protein drug stability: a formulation challenge. Nat Rev Drug Disc.

[2] Den Engelsman J, Garidel P, Smulders R, Koll H, Smith B, Bassarab S, Seidl A, Hainzl O, Jiskoot W (2011). Strategies for the assessment of protein aggregates in pharmaceutical biotech product development. Pharm Res.

[3] Manning RR, Holcomb RE, Wilson GA, Henry CS, Manning MC (2014). Review of orthogonal methods to SEC for quantitation and characterization of protein aggregates. Biopharm Int.

[4] Carpenter JF, Randolph TW, Jiskoot W, Crommelin DJA, Middaugh CR, Winter G (2010). Potential inaccurate quantitation and sizing of protein aggregates by size exclusion chromatography: essential need to use orthogonal methods to assure the quality of therapeutic protein products. J Pharm Sci.

[5] USFDA (2014) Guidance for industry immunogenicity assessment for therapeutic protein products.

[6] Wang W, Singh SK, Li N, Toler MR, King KR, Nema S (2012). Immunogenicity of protein aggregates—concerns and realities. Int J Pharm.

[7] Barrett KE, Brooks H, Boitano S, Barman SM (2010). Ganong’s review of medical physiology.

[8] Gonzalez-Quintela A, Alende R, Gude F, Campos J, Rey J, Meijide LM, Fernandez-Merino C, Vidal C (2008). Serum levels of immunoglobulins (IgG, IgA, IgM) in a general adult population and their relationship with alcohol consumption, smoking and common metabolic abnormalities. Clin Exp Immunol.

[9] Pretlow TG, Pretlow TP (1983). Cell separation: methods and selected applications.

[10] Pitt WG, Alizadeh M, Husseini GA, McClellan DS, Buchanan CM, Bledsoe CG, Robison RA, Blanco R, Roeder BL, Melville M, Hunter AK (2016). Rapid separation of bacteria from blood—review and outlook. Biotechnol Prog.

[11] Wahlund KG, Nilsson L, Williams SKR, Caldwell K (2012). Flow FFF—basics and key applications. Field-flow fractionation in biopolymer analysis.

[12] Litzén A, Wahlund KG (1991). Zone broadening and dilution in rectangular and trapezoidal asymmetrical flow field-flow fractionation channels. Anal Chem.

[13] Wahlund KG, Litzén A (1989). Application of an asymmetrical flow field-flow fractionation channel to the separation and characterization of proteins, plasmids, plasmid fragments, polysaccharides and unicellular algae. J Chromatogr.

[14] Qureshi RN, Kok WT (2011). Application of flow field-flow fractionation for the characterization of macromolecules of biological interest: a review. Anal Bioanal Chem.

[15] Rambaldi DC, Reschiglian P, Zattoni A (2011). Flow field-flow fractionation: recent trends in protein analysis. Anal Bioanal Chem.

[16] Nilsson L (2013). Separation and characterization of food macromolecules using field-flow fractionation: a review. Food Hydrocoll.

[17] Choi J, Lee S, Linares-Pastén JA, Nilsson L (2018). Study on oligomerization of glutamate decarboxylase from Lactobacillus brevis using asymmetrical flow field-flow fractionation (AF4) with light scattering techniques. Anal Bioanal Chem.

[18] Cragnell C, Choi J, Segad M, Lee S, Nilsson L, Skepö M (2017). Bovine β-casein has a polydisperse distribution of equilibrium micelles. Food Hydrocoll.

[19] Sandra K, Vandenheede I, Sandra P (2014). Modern chromatographic and mass spectrometric techniques for protein biopharmaceutical characterization. J Chromatogr A.

[20] Shin K, Choi J, Cho JH, Yoon MY, Lee S, Chung H (2015). Feasibility of asymmetrical flow field-flow fractionation as a method for detecting protective antigen by direct recognition of size-increased target-captured nanoprobes. J Chromatogr A.

[21] Litzén A, Walter JK, Krischollek H, Wahlund KG (1993). Separation and quantitation of monoclonal antibody aggregates by asymmetrical flow field-flow fractionation and comparison to gel-permeation chromatography. Anal Biochem.

[22] Madörin M, Van Hoogevest P, Hilfiker R, Langwost B, Kresbach GM, Ehrat M, Leuenberger H (1997). Analysis of drug/plasma protein interactions by means of asymmetrical flow field-flow fractionation. Pharm Res.

[23] Li P, Giddings JC (1996). Isolation and measurement of colloids in human plasma by membrane-selective flow field-flow fractionation: lipoproteins and pharmaceutical colloids. J Pharm Sci.

[24] Park I, Paeng KJ, Yoon Y, Song JH, Moon MH (2002). Separation and selective detection of lipoprotein particles of patients with coronary artery disease by frit-inlet asymmetrical flow field-flow fractionation. J Chromatogr B Anal Technol Biomed Life Sci.

[25] Dati F, Schumann G, Thomas L, Aguzzi F, Baudner S, Bienvenu J, Blaabjerg O, Blirup-Jensen S, Carlstrom A, Petersen PH, Johnson AM, Milford-Ward A, Ritchie RF, Svendsen PJ, Whicher J (1996). Consensus of a group of professional societies and diagnostic companies on guidelines for interim reference ranges for 14 proteins in serum based on the standardization against the IFCC/BCR/CAP Reference Material (CRM 470). Eur J Clin Chem Clin Biochem.

[26] Abbas AK, Lichtman AH, Pober JS (2000). Cellular and molecular immunology.

[27] Caldwell KD, Nguyen TT, Myers MN, Giddings JC (1979). Observations on anomalous retention in steric field-flow fractionation. Sep Sci Technol.

[28] Williams PS, Giddings JC (1994). Theory of field programmed field-flow fractionation with corrections for steric effects. Anal Chem.

[29] Bee JS, Goletz TJ, Ragheb JA (2012). The future of protein particle characterization and understanding its potential to diminish the immunogenicity of biopharmaceuticals: a shared perspective. J Pharm Sci.

