# Supplementary material for: Proteins and antibodies in serum, plasma, and whole blood—size characterization using asymmetrical flow field-flow fractionation (AF4)
Source: Anal Bioanal Chem. 2018 May 29;410(20):4867–73. doi: 10.1007/s00216-018-1127-2 (PMC6061777; doi:10.1007/s00216-018-1127-2)
Supplement: Supplementary file 1 — (PDF 127 kb) [file 216_2018_1127_MOESM1_ESM.pdf]

Analytical and Bioanalytical Chemistry

Electronic Supplementary Material

Proteins and antibodies in serum, plasma and whole blood – size characterization using asymmetrical flow field-flow fractionation (AF4)

Mats Leeman, Jaeyeong Choi, Sebastian Hansson, Matilda Ulmius Storm, Lars Nilsson

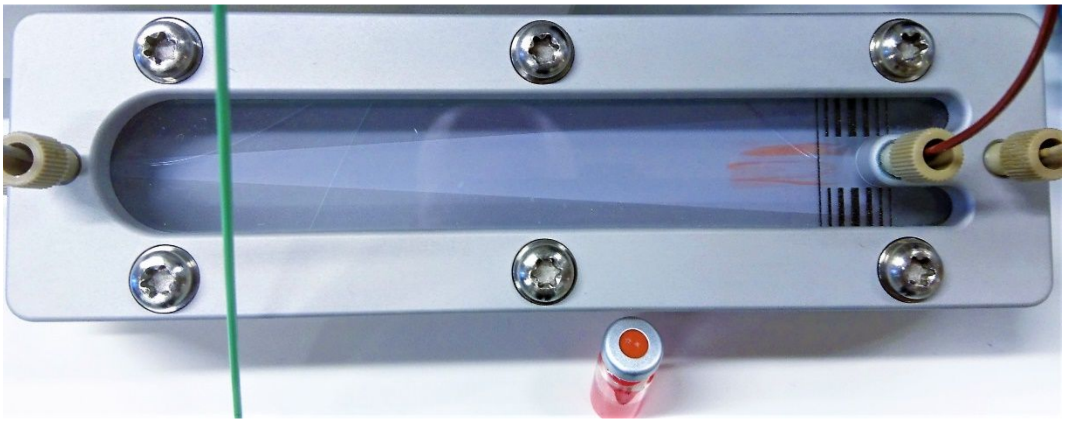

Fig. S1 Photograph of the AF4 channel while analysing the whole blood. The red colour seen in the channel is the blood that is immobilized on the channel. Photograph taken while in elution mode
